# A conversation on the effects of the COVID-19 pandemic on junior researchers’ careers with funders and university leaders

**DOI:** 10.1038/s41467-021-22040-3

**Published:** 2021-04-07

## Abstract

The various restrictions applied across the globe to contain the COVID-19 pandemic have been impacting the way we knew how to work. Dr. Matthews (a scientific program manager at the National Institute of Neurological Disorders and Stroke—NINDS), Dr. David del Álamo Rodriguez (head of the European Molecular Biology Organization—EMBO—fellowship program), and Dr. Gray (Associate Dean for the Sciences at the Advanced Science Research Center of the City University of New York) shared with *Nature Communications* their thoughts on how funders and university leadership can support early career researchers and young faculty through the COVID-19 pandemic.

Please tell us a bit about your research background and position.

My name is **Marguerite Matthews**. I’m a scientific program manager at the National Institute of Neurological Disorders and Stroke (NINDS) in the Office of Programs to Enhance Neuroscience Workforce Diversity. In this role, I support the Institute’s diversity initiatives and programs that provide neuroscience research training and career development for underrepresented trainees and early career investigators. In particular, I help manage Research Supplements to Promote Diversity and Re-entry in Biomedical Research, the NRSA Individual Predoctoral Fellowships to Promote Diversity in Health-Related Research (F31), the NINDS Neuroscience Development for Advancing the Careers of a Diverse Research Workforce (R25), and the NIH Blueprint Program for Enhancing Neuroscience Diversity through Undergraduate Research Education Experiences (BP-ENDURE, R25).

**Figure 1:**
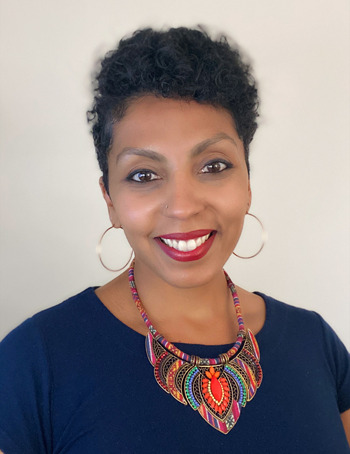
Dr. **Marguerite Matthews**, scientific program manager, National Institute of Health.

My name is **David del Álamo Rodriguez**. I obtained my PhD in Madrid, Spain, in 2003 working on Drosophila developmental genetics. After two postdocs at the Mount Sinai School of Medicine and the Pasteur Institute in New York and Paris, respectively, I moved away from active research and became a scientific editor for The European Molecular Biology Organization (EMBO) Journal in 2011. I have been the head of the EMBO Fellowship Programme since 2016. This programme awards about 180 postdoctoral and about 300 short-term fellowships each year.

**Figure 2:**
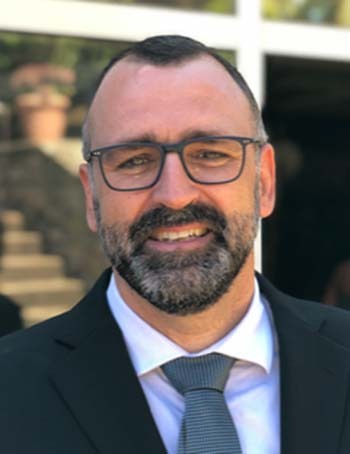
Dr. **David del Álamo Rodriguez**, head of the European Molecular Biology Organization (EMBO) Fellowship Programme.

I’m **Annette Gray**. I studied neuroscience at Brown University and Brandeis University. I then shifted my career to supporting the research and education of others. In my current role, as Associate Dean for the Sciences at the Advanced Science Research Center for the Graduate Center of the City University of New York (ASRC CUNY), I work with all levels of researchers at one of the nation’s largest and most diverse public universities to facilitate and amplify interdisciplinary STEM research. I oversee labs and shared core facilities, sponsored programs, scientific and public events and programs, and catalyze research and educational collaborations across CUNY. Since March, my effort has focused on maintaining the CUNY ASRC as a safe place for research during the pandemic to ensure that our Early Career Researchers (ECRs) are able to make as much progress as possible without worry for their health.

**Figure 3:**
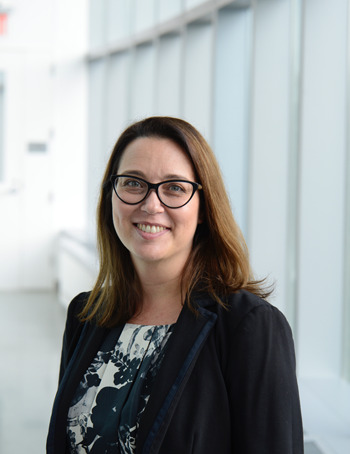
Dr. **Annette Gray**, Associate Dean, Sciences at the Advanced Science Research Center for the Graduate Center of the City University of New York (ASRC CUNY). Alex Irklievski

What are your main concerns regarding the long-term impact of COVID-19 on the research and career progression of junior scientists?

**MM:** In addition to the devastating loss of over one million lives worldwide, COVID-19 is clearly taking a toll on our way of life, economic and financial stability, and mental wellness. We have not even begun to understand the full impact that COVID-19 will have on the health of those recovering from the disease and we certainly cannot predict how the pandemic will impact society more broadly, even after vaccines are made widely available and we are able to resume life without social distancing. Such uncertainty is weighing heavily on research training—both in terms of the increased difficulty in physically conducting research studies and the mental acuity and motivation needed to be a productive scientist. It seems cruel to expect that trainees and early career investigators carry on business as usual in the midst of a pandemic that is showing few signs of slowing down, in the US in particular, but it is also necessary to keep making some forward progress. It is my hope that junior scientists have the resources and support—from friends, family, peers, research mentors, faculty, program officers, mental health professionals – they need to stay encouraged and equipped to continue along their career trajectories.

**DdAR:** We [at EMBO] are aware of specific issues suffered by scientists in situations that have been complicated by the pandemic, for instance, scientists moving to different countries with their families, or those being unable to start new positions due to widespread lockdowns. These are groups of scientists affected by the COVID-19 pandemic much more than scientists in more stable situations. Said that, if we consider the community of junior scientists at large the long-term impact will most likely be rather limited. There will be some career progression delays and associated funding problems, but researchers’ careers will most likely not be crucially affected in the long run. The scientific career in academia is a long-term enterprise that is usually not defined by a short period of difficulties.

I’m also aware that scientists in countries where the pandemic is more difficult to contain and where research funding are more limited than in other countries will suffer harsher consequences.

**AG:** The pandemic’s impact is not uniform across all individuals, and I hope universities enact policies that recognize this fact. For instance, as early as May, it was clear that working from home during the lockdown has had a disproportionately negative impact on women’s productivity, as measured by authorship on preprints and involvement in new projects (see article^1^.). An analysis on COVID-19 effects on academic productivity shows that, compared to the same months in 2019, men scientists have increased their preprint resubmission more than their women colleagues (resource available here). For postdoctoral fellows about to hit the academic job market or untenured junior faculty competing for their first grants and awards, even a small difference in their productivity could influence their ability to succeed. Hiring committees should discuss and be aware of differential impacts of the pandemic. Universities should also keep in mind that, before the pandemic, gender-neutral family leave policies had not benefited women as much as hoped because of the underlying differential impact of raising children.

How are funding bodies and academic institutions responding to the crisis right now, and how is the ongoing COVID-19 pandemic affecting the funding landscape in the mid- and long-term? What are the long-term plans of funding bodies and academic institutions to support young researchers through the pandemic?

**MM:** The National Institutes of Health (NIH) is deeply concerned for the health and safety of people involved in NIH research, and the effects COVID-19 has had and continues to have on the biomedical enterprise. NIH wants to assure our recipient community that NIH is working to help researchers continue their research. For more information on guidance and information regarding NIH applicants and recipients of NIH funding, visit the NIH dedicated page.

**DdAR:** Many funding agencies adopted measures already in March and April to support scientists. EMBO was one of the first organizations to extend the duration of all granted (EMBO) postdoctoral fellowships by 2 months. This initiative was quickly followed by other funding bodies. We also extended lab visits funded by the EMBO short-term fellowships and provided these fellows with additional funding to cover travel and living expenses. However, no budget is ever designed to respond to an event of the magnitude we are facing. All funding bodies understand that our responses were probably insufficient to offset all the immediate effects of the COVID-19 pandemic, but available funds did not allow for further actions beyond what I have described above. We also eased eligibility criteria to the extent allowed by budgets and internal regulations. We have been monitoring the situation, retained close contact with researchers and have tried to adapt to an ever-changing landscape, but there is only so much funding agencies can do. Collaboration at all levels, including national science programmes, research institutions and even direct supervisors is required to support ECRs.

**AG:** The need for increased public scientific literacy and acceptance has never been more apparent. Having to forego in-person public outreach forced us to create virtual programs that give teachers and parents the flexibility to work them into virtual and hybrid learning schedules. We also transitioned our Community Sensor Lab online, which trains students how to build simple DIY environmental sensors, and are expanding this program to inspire a love of STEM through simple, research exercises that allow anyone to collect and anlyze data at home.

We obviously increased IT support, including added hardware and software. Faculty from the CUNY ASRC also led online conferences. Such efforts ensured that junior scientists could still share their research, network with scientists, and form new collaborations. They also ensured that more people could attend, at lower cost, not to mention in a more sustainable platform for the environment.

Importantly, public universities are struggling because of uncertain federal, state and city budgets, which impacts admissions for next fall and recruitment and retention of faculty, postdocs, and other research staff. Budget cuts may outlast the pandemic and stretch out for two or more years, endangering the important niche that public universities fill in higher education. We’d like to see more philanthropic donors make compensatory investments. CUNY educates 500,000 students each year, and over 50% of CUNY students are Black and/or LatinX. Fourteen of its colleges rank in the top tier of US colleges in terms of improving socioeconomic mobility. CUNY aims to expand workforce development and certificate programs to help New Yorkers retool and upskill to improve their employment options. But all of this is at risk if funding is not in place.

In your opinion, what can supervisors, mentors, university leadership, and/or funding bodies do to support ECRs and junior faculty during lockdowns and post-pandemic?

**MM:** Everyone must be willing to apply patience, empathy, flexibility, and grace to one another during this pandemic and beyond. As a rule of thumb, it is incredibly important to communicate effectively with funding agency staff about your challenges and needs and how you think they can support you. It is the responsibility of applicants and grantees to communicate their situations (whether pandemic-related or not) to their program officers and/or grant management staff for guidance and flexibility where possible. We, as funding agency staff, cannot help you or provide you with relevant resources if we don’t know what’s going on.

**DdAR:** The most important aspect in my opinion is flexibility. It should be exercised at all levels in research institutions, from supervisors, who have to understand the especially difficult circumstances their team members are facing, to top level leadership, who must support and care about the well-being of their staff when working in isolation. Special attention should be given to those at the beginning or the end of their contracts with the institution. They may need extra support, sometimes requiring contract extensions. Naturally, mentoring or help with creating new networks are important, but probably not more so now than they are under usual circumstances. We have found that the administrative burden has increased on junior researchers, partly due to the fact that administrative personnel have been working remotely for the better part of 2020. The COVID-19 pandemic has affected the way researchers work. Some changes are positive, for instance the opportunity to connect more at global level through e-conferences (as reported in this article^2^.). This is probably a good opportunity to transition to a fully electronic system that again adds flexibility to procedures. Nevertheless, the pandemic has unequally affected researchers, too (as reported in this article^3^.).

**AG:** Senior faculty and administrators must make added effort to support diverse young scientists proactively. For recruitment and promotion, evaluations of ECRs should be more nuanced, and consider candidates’ personal paths, to ensure that the best and brightest are appropriately identified. Universities could also help the most impacted ECRs by providing relief from teaching and administrative obligations to give them more time to get their research back on track, and prioritize them for internal awards and nominations for limited competition awards.

Further, dozens of articles, including from the Student Experience in the Research University Consortium based at the University of California – Berkeley, have been published on the surge in mental health issues among graduate students and postdocs during the pandemic. With the fear of an even more challenging job market and increased feelings of isolation and a lack of productivity associated with the COVID-19 pandemic, I am concerned we will lose many excellent researchers, especially among vulnerable groups, such as women and underrepresented groups in STEM. Universities should tirelessly inform faculty, students, and staff about mental health and wellness resources, and create opportunities for researchers who feel isolated to form peer support groups, being mindful of equity.

Do you think the pandemic is exacerbating/will exacerbate issues associated with diversity and inclusion in academia? If so, how? In your opinion, what can be done to prevent this?

**MM:** One of the best outcomes of this pandemic and resurgence of the Black Lives Matter movements has been the incredible community building. To witness the rise of #BlackInNeuro, #BlackInMicro, #BlackInGenetics, #BlackInCancer, #BlackInChem, etc has been so inspiring. While many groups have historically been shut out of or discouraged from pursuing scientific training and careers, individuals from these groups have used this new “virtual reality” as an opportunity to rally together across the globe to share their love of their science(s), uplift each other, and commiserate over shared experiences—good and bad. Universities, national organizations, funding institutions, and individuals are taking notice of these communities and the demand for inclusive, welcoming environments, and are working to answer the call to better address diversity, equity, and inclusion in academia and other sectors of the workforce. Now is the time for us all to do the hard work of dismantling the barriers and biases that keep us from embracing diverse thoughts, ideas, and perspectives needed to bring scientific and technological discoveries to their fullest potential.

**DdAR:** As with any other emergency -financial, health-related or of any other type- the less privileged are affected the most. Scientists who have access to good funding, attend excellent universities, or work a world-leading research institutions will probably suffer less comparatively. This disparity also applies to scientists in particularly difficult situations even before the COVID-19 pandemic started, such as those having to take care of other family members or facing a health condition. For instance, according to recent surveys (as reported in this article^3^.), women (and particularly women with children) saw a significant reduction in their time devoted to work during the first few months of the pandemic due to care givers’ duties. Again, monitoring closely the situation of scientists and being more flexible with those who are having a more difficult time will be key to avoid extending the gap in certain areas. Direct effects of the pandemic on other inclusion or diversity issues are more difficult to ascertain at this point and will probably require a more detailed and longer-term analysis.

**AG:** Latinx and Black high school students were far more likely to report changing higher education plans, such as attending less expensive schools closer to home or attending community college instead of a 4-year college, compared to their white peers (as reported in this article). At home and hybrid learning for K-12 students also are more likely to leave children from minority groups and lower socioeconomic backgrounds, who have less access to regular distance learning activities, even further behind (a few articles can be found here, and here). As with health impacts, educational impacts of the pandemic ride a top pre-existing disparities.

We also can’t forget that 50% of doctoral students and postdocs in STEM are internationals, whose plans to work in the US have been delayed or cut short. Altogether, we have to double down on our initiatives to promote diversity, equity, and inclusion to limit the damage of the COVID-19 pandemic to young researchers’ careers because we can only reach the greatest heights of discovery and the greatest depths of understanding when we embrace and lift all voices.

Other thoughts?

**DdAR:** We have to keep in mind that large scale analyses on the effects of the COVID-19 pandemic on early career researchers may lead to overlooking individual suffering. A theoretical physicist or computational biologist without any dependents that faced the lockdowns in March/April in the middle of their thesis/postdoctoral period probably saw a minor effect of the pandemic on their work routine and research output compared to wet lab researchers having to take care of their families or those finishing their contracts and having to find new jobs. Keeping open communication lines with scientists and evaluating every case according to its particular circumstances has been and will continue to be crucial at least for the best part of 2021.

**AG:** Personally, I admit—even though I count myself very lucky to have stayed healthy, for my family to be healthy—getting through the pandemic has been an incredible struggle. Some days, I am productive, but some days are a slog. But after each day, there is another new day that could be brighter. This makes me confident that we have an opportunity to “build back better” to steal a slogan, and I hope to see conversations and action across university boundaries and across STEM sectors that address these issues systematically and not in isolated siloes.
